# Cronyn’s Boudoir Medicines

**Published:** 1878-02

**Authors:** 


### CRONYN'S

### BOUDOIR MEDICINES,

FOR ALL

### DISEASES PECULIAR TO FEMALES.

**Recommended and Endorsed by the Medical Faculty, and for Sale by all Respectable Druggists.**

---

### What Cronyn's BOUDOIR MEDICINES Will Prevent and Cure.

AMENORRHŒA *or "absence of the monthlies;"* ABSCESSES; DISPLACEMENTS OF THE WOMB; DRIBBLING OF URINE; DROPSY; DYSMENORRHŒA, *or "painful monthlies;"* CHRONIC ENLARGEMENT AND INFLAMMATION OF WOMB; LEUCORRHŒA, *or "Whites;"* MENORRHAGIA, *or an excessive flow during the "monthlies,"* METORRHAGIA, *or flowing between times;* HYSTERIA AND NERVOUSNESS; OVARIAN IRRITATION; POLYPUS; PROLAPUS, *or falling of womb;* PRURITUS VULVA, *or itching at entrance of vagina;* TUMORS OF THE WOMB; OVARIAN TUMORS; PERIODIC INSANITY *from Uterine Disease.*

Many cases of Cancer have been benefited by the *BOUDOIR MEDICINES.*

Recommended by Prof. T. Gillard Thomas, M. D., Prof. A. F. A. King, M. D., Prof. W. H. Byford, M. D., Prof. D. W. Bicknell, M. D., Prof. James P. White, M. D.

---

*All Communications* and money orders should be addressed to WILLIAM CRONYN, M. D., Dunkirk, N. Y.

PECULIAR OR COMPLICATED CASES—Any lady unable to consult me personally may do so by letter, giving a plain statement of her ills, their origin, cause, if known, present symptoms, etc. State all in confidence, withholding nothing that can enable me to judge of the nature or severity of the disease. *All correspondence is held Inviolably Sacred.* All such will receive promptly in return, a letter giving my opinion both as to the nature of her disease and the length of treatment necessary to effect a full and permanent cure. Letters of consultation should always be accompanied by a fee of one dollar.

Dr. Cronyn *was* a regular physician, but seems to have sold his birthright for "Boudoir."

The distinguished physicians quoted as endorsing and recommending "Boudoir," will probably tell us what it is; they must be in the secret.*

We are, of late, frequently asked: "What is *Boudoir?*" Not knowing much about it, we have to answer, of course, one of the new remedies.

